# The Use and Perceptions of AI Chatbots in Medical Research: An International Cross-Sectional Survey

**DOI:** 10.7759/cureus.100908

**Published:** 2026-01-06

**Authors:** Hassan M Alturaiki, Mujtaba M Al Khamees, Hassan A Alradhi, Ohud T Alharbi, Fatma Kefi, Ghadeer A Alali, Fadila Bello, Talha Shahzad, Abdullah Husain H Al Ramadan

**Affiliations:** 1 Department of Medicine, College of Medicine, King Faisal University, Hofuf, SAU; 2 Department of General Surgery, King Fahad Hospital, Hofuf, SAU; 3 Department of Neurology, King Faisal University, Hofuf, SAU; 4 Department of Neurosurgery, King Fahad Medical City, Riyadh, SAU; 5 Medical School, Batterjee Medical College, Jeddah, SAU; 6 College of Clinical Pharmacy, Imam Abdulramana Bin Faisal University, Khobar, SAU; 7 Faculty of Medicine, Aminu Kano Teaching Hospital, Kano, NGA; 8 Department of Emergency Medicine, University of Central Lancashire, Preston, GBR; 9 Department of Neurosurgery and Spine Surgery, Qatif Central Hospital, Qatif, SAU

**Keywords:** artificial intelligence, chatbot, chat gpt, large language models, medical research

## Abstract

Background

Artificial Intelligence (AI) has undergone remarkable progress, leading to the development of advanced large language models (LLMs). Despite these LLMs' growing adoption, concerns persist regarding the scientific accuracy of AI-generated content, and their acceptance within academic publishing remains contentious. This study aimed to describe AI-chatbot use patterns and to assess medical researchers’ perceptions of impact on research credibility, ethical concerns, guideline awareness, and disclosure of future intentions.

Methodology

A cross-sectional survey-based study spread into Saudi Arabia, Nigeria, Tunis, and England from 2023 to 2024 surveyed researchers, excluding non-medical and non-publishing researchers.

Results

We analyzed 434 respondents; 175 (40.3%) reported AI-chatbot use. Use varied by country (32.8%-45.9%), but neither gender nor country was significantly associated with use. Older age and more senior roles were associated with lower odds of use (odds ratio (OR): ages 41-50 years, 0.32; residents, 0.31; consultants, 0.17; *P* ≤ 0.009). Awareness strongly predicted use (OR 15.53), as did guideline awareness (OR 2.47), trust (*P* = 0.005), hypothesis formation (*P* = 0.001), willingness to cite (*P* = 0.003), and future use (*P* < 0.001); intention to declare use during submission did not differ (*P* = 0.468).

Conclusions

Our study shows that medical researchers have a positive attitude toward using AI chatbots, but with ethical and accuracy concerns requiring further interventions to create systematic unified rules.

## Introduction

Artificial Intelligence (AI), first introduced in 1956, refers to the ability of computer systems to perform tasks typically requiring human intelligence. This field has seen significant advancements, particularly with the development of AI-driven large language models (LLMs) such as Google Bard, Gemini, Bing AI, and Chat Generative Pretrained Transformer (ChatGPT), developed by OpenAI, which is designed to generate responses that are similar to those a human might provide [1]. The presence of ChatGPT and similar services has significantly expanded the range of available online text generation tools [2]. Assisting in the scientific community, in tasks such as conducting literature reviews, developing outlines, enhancing writing style, summarizing and analyzing data, identifying key findings, and citing references [2-4]

Many guidelines for authors regarding the use of AI chatbots have been provided for researchers. However, the acceptance has been a topic of controversial perspectives, such as in academic publishing, with some high-impact journals, such as Springer-Nature and Science, not accepting ChatGPT as a coauthor, while others, like many Elsevier journals, allow its use with disclosure [2,5,6]. Studies highlight both promise and limitations: ChatGPT-generated texts often score lower on plagiarism checks and produce coherent writing, yet semantic errors, fabricated references, and concerns about accuracy and ethics persist [7-11]. For example, a survey of urologists found that nearly half reported using ChatGPT in academic or clinical tasks, even though 62.2% expressed potential ethical concern [12]. Accordingly, this study aimed to describe the prevalence and patterns of AI chatbot use among medical researchers, assess researchers’ perceptions regarding AI chatbots’ influence on research activities and scientific credibility, and explore researchers’ ethical concerns, awareness of relevant guidelines, and attitudes toward future use and disclosure of AI chatbot use in medical research and manuscript submission.

## Materials and methods

Study design and setting

We conducted an observational, cross-sectional survey to assess the use and perceptions of AI chatbots among medical researchers. The survey was administered online and targeted participants in multiple countries (Saudi Arabia, Nigeria, Tunisia, and the United Kingdom) to provide an international perspective. Data collection took place between December 2023 and January 2024. The questionnaire was in English and self-administered, allowing respondents from various cities and institutions to participate remotely.

Participants and recruitment

Eligible participants were medical researchers who (1) had either published at least one study or were currently involved in a medical research project, and (2) resided in Saudi Arabia, Nigeria, Tunisia, or the United Kingdom (England), regardless of nationality. Individuals were excluded if they had never conducted or contributed to a research project, if they were outside the mentioned countries, or if they were not in the medical field. Recruitment was done via social media apps, including X platform, WhatsApp, Telegram, LinkedIn, Snapchat, and Instagram.

Data collection and variables

Data were collected using a structured, self-administered English-language online questionnaire developed for this study and distributed via social media platforms. The instrument was not pilot tested and did not undergo formal validation; therefore, all perception/attitude findings should be interpreted as exploratory. The survey instrument included the following sections: (a) Screening questions, confirming the inclusion and exclusion criteria. (b) Demographics, including participant characteristics such as age, gender, country of residence, and professional role. (c) AI Chatbot awareness and use, including questions on whether the participant was aware of AI chatbots for research before the survey, and whether they had ever used such tools in their own research. For those who had used AI chatbots, additional items asked which chatbot was used and the participant’s level of satisfaction with the experience. (d) Perceived impact on research, using a Likert scale for transforming subjective responses into quantifiable data, including items from 1 = “significantly worsens” to 5 = “significantly improves”, asking how the participant believes AI chatbots affect various aspects of medical research [13]. (e) Ethical perspectives include questions about ethical considerations of AI chatbot use in research, such as whether there should be ethical guidelines or regulations, and opinions on whether incorporating AI-generated text into a manuscript constitutes plagiarism. Participants answered these on categorical scales, e.g., Yes/No/Maybe. Finally, (f) Future intentions and attitudes include items gauging the participants’ attitudes toward AI chatbots and their willingness to use or recommend these tools in future research endeavors (Appendices A-F).

Primary outcome variables in this survey included the self-reported use of AI chatbots in research (binary: yes or no) and the perception of AI chatbots’ impact on research (measured by the Likert-scale ratings across various aspects of research productivity and quality). Additional outcome measures were participants’ ethical stances (e.g., whether they believe guidelines are needed) and future intentions regarding AI chatbot use. Key explanatory variables (potential predictors of differences in outcomes) were the participants’ demographic characteristics, such as age group, gender, country, and professional role. We did not explicitly designate any confounders or effect modifiers a priori; however, we recorded a range of participant characteristics to allow exploratory subgroup analyses for any factors that might influence responses.

Statistical analysis and sample size

**Figure 1:**
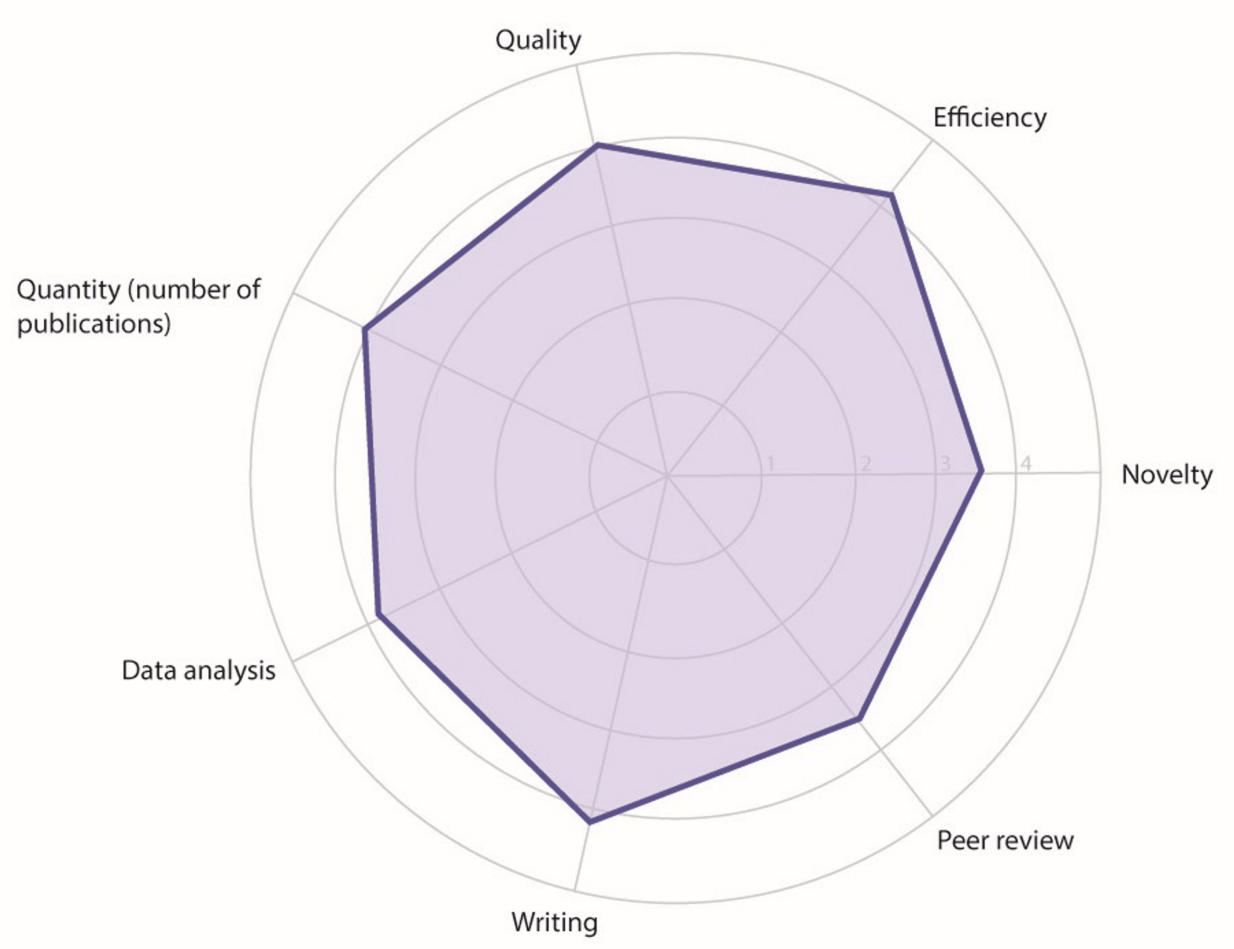
Study participants’ perspectives on the impact of AI chatbots in medical research. A radar plot illustrating the average response for each aspect. A score of 1 corresponds to significantly worsens, 2 to worsens, 3 to neutral, 4 to improves, and 5 to significantly improves.

Analyses were performed in IBM SPSS (Windows version 2021; IBM Corp., Armonk, NY), with data handling in Microsoft Excel 2021. Figure 1 was created by the authors to summarize the sample and survey responses (counts and percentages for categorical variables; stacked distributions for the impact items. Bivariate associations comparing the proportion of AI chatbot users across subgroups (e.g., region, gender, age group, profession, and attitude/perception categories) were tested using Pearson’s chi-square (χ²), and crude odds ratios (ORs) with 95% confidence intervals were reported relative to prespecified reference categories in Table 2. The final sample size was determined by the number of eligible respondents who completed the survey during the study period (434 participants). No formal priori calculation was performed, through convenience sampling.

## Results

Participants' characteristics

We analyzed 434 respondents after excluding 68 participants; 175 (40.3%) reported having used an AI chatbot in their research. By country, nearly half were from Saudi Arabia (216, 49.8%), followed by Nigeria (90, 20.7%), the United Kingdom (67, 15.4%), and Tunisia (61, 14.1%). Within countries, the proportion of respondents who had used an AI chatbot ranged from 22 (32.8%) in the United Kingdom to 28 (45.9%) in Tunisia; Saudi Arabia and Nigeria reported use in 92 (42.6%) and 33 (36.7%) of respondents, respectively, as shown in Table 1.

**Table 1:** Sociodemographic data of the study’s participants.

Items		Did not use an AI chatbot, *n* (%)	Used an AI chatbot, *n* (%)	Total *N*
Country	Saudi Arabia	124 (57.41%)	92 (42.59%)	216
	Nigeria	57 (63.33%)	33 (36.67%)	90
	United Kingdom	45 (67.16%)	22 (32.84%)	67
	Tunisia	33 (54.10%)	28 (45.90%)	61
Gender	Female	125 (61.58%)	78 (38.42%)	203
	Male	134 (58.01%)	97 (41.99%)	231
Age group (years)	18-30	171 (55.16%)	139 (44.84%)	310
	31-40	55 (66.27%)	28 (33.73%)	83
	41-50	27 (79.41%)	7 (20.59%)	34
	50+	6 (85.71%)	1 (14.29%)	7
Profession	Students	77 (46.11%)	90 (53.89%)	167
	Interns	39 (62.90%)	23 (37.10%)	62
	Pharmacists	11 (61.11%)	7 (38.89%)	18
	Healthcare workers	8 (66.67%)	4 (33.33%)	12
	Residents	74 (73.27%)	27 (26.73%)	101
	Specialists	12 (44.44%)	15 (55.56%)	27
	Consultants	30 (83.33%)	6 (16.67%)	36
	Academics	8 (72.73%)	3 (27.27%)	11

Demographic differences

Age and professional seniority were the key demographic correlates of use. Compared with 18-30 year olds, the odds of being a user of an AI chatbot in medical research were lower for 41-50 year olds (OR = 0.32, 95% CI 0.13-0.75) and trended lower for 31-40 year olds (OR = 0.63, 95% CI 0.38-1.04; *P* = 0.009). Students formed the reference group for profession; interns showed modestly lower use (OR = 0.50, 95% CI 0.28-0.92), residents/general practitioners (GPs) substantially lower use (OR = 0.31, 95% CI 0.18-0.53), and consultants the lowest use (OR = 0.17, 95% CI 0.07-0.43; *P* < 0.001). Gender (*P* = 0.450) and country (*P* = 0.346) were not significantly associated with use (Table 2).

**Table 2:** Bivariate associations of AI chatbot use (users vs non-users) with demographics, awareness, perceptions, and attitudes; crude ORs (95% CI). AI, artificial intelligence; OR, odds ratio; CI, confidence interval; ref, reference category; GP, general practitioner; *N*, number

Variable (overall *P*)	Category	Users (*N*)	Non-users (*N*)	Users in category (%)	Crude OR vs. ref. (95% CI)
Age (years) (*P* = 0.009)	18-30 (ref)	139	171	44.8%	-
	31-40	28	55	33.7%	0.63 (0.38-1.04)
	41-50	7	27	20.6%	0.32 (0.13-0.75)
	50+	1	6	14.3%	0.21 (0.02-1.72)
Gender (*P* = 0.450)	Male (ref)	97	134	42.0%	
	Female	78	125	38.4%	0.86 (0.59-1.27)
Country (*P* = 0.346)	Saudi Arabia (ref)	92	124	42.6%	-
	The United Kingdom	22	45	32.8%	0.66 (0.37-1.17)
	Tunisia	28	33	45.9%	1.14 (0.65-2.02)
	Nigeria	33	57	36.7%	0.78 (0.47-1.29)
Profession (*P* < 0.001)	Student (ref)	90	77	53.9%	-
	Intern	23	39	37.1%	0.50 (0.28-0.92)
	Resident/GP	27	74	26.7%	0.31 (0.18-0.53)
	Specialist	15	12	55.6%	1.07 (0.47-2.42)
	Consultant	6	30	16.7%	0.17 (0.07-0.43)
	Academics	3	8	27.3%	0.32 (0.08-1.25)
	Pharmacist	7	11	38.9%	0.54 (0.20-1.47)
	Healthcare workers	4	8	33.3%	0.43 (0.12-1.48)
Aware of using an AI chatbot (*P* < 0.001)	No (ref)	4	69	5.5%	-
	Yes	171	190	47.4%	15.53 (5.55-43.44)
Sources of the awareness about an AI chatbot (*P* = 0.073)	Social media (ref)	83	105	44.1%	-
	Friends	61	49	55.5%	1.57 (0.98-2.53)
	News	6	13	31.6%	0.58 (0.21-1.60)
	Conferences	7	12	36.8%	0.74 (0.28-1.96)
	Other	13	25	34.2%	0.66 (0.32-1.36)
Concerns	Accuracy of data (ref) (*P* = 0.044)	109	136	44.5%	-
	Ethical concerns (*P* = 0.121)	82	141	36.8%	0.73 (0.50-1.05)
	Data privacy (*P* = 0.062)	41	82	33.3%	0.62 (0.40-0.98)
	Lack of human oversight (*P* = 0.830)	86	130	39.8%	0.83 (0.57-1.20)
	Bias in AI algorithms (*P* = 0.269)	81	106	43.3%	0.95 (0.65-1.40)
	Others (*P*= 0.063)	12	32	27.3%	0.47 (0.23-0.95)
Believe the need for ethical considerations (*P* = 0.175)	No (ref)	16	14	53.3%	-
	Maybe	42	53	44.2%	0.69 (0.30-1.58)
	Yes	117	192	37.9%	0.53 (0.25-1.13)
Awareness of existing regulatory guidelines (*P* < 0.001)	No (ref)	109	208	34.4%	-
	Yes	66	51	56.4%	2.47 (1.60-3.81)
Perception about AI-generated text being plagiarism (*P* = 0.026)	No (ref)	49	46	51.6%	-
	Maybe	75	138	35.2%	0.51 (0.31-0.83)
	Yes	51	75	40.5%	0.64 (0.37-1.09)
Trust of AI chatbot (*P* = 0.005)	Completely do not trust (ref)	2	9	18.2%	-
	Mostly do not trust	28	47	37.3%	2.68 (0.54-13.31)
	Neutral	77	144	34.8%	2.41 (0.51-11.42)
	Mostly trust	64	54	54.2%	5.33 (1.10-25.75)
	Completely trust	4	5	44.4%	3.60 (0.48-27.11)
Trust the result of studies that used an AI chatbot (*P* = 0.015)	Completely do not trust (ref)	3	7	30.0%	-
	Mostly do not trust	22	52	29.7%	0.99 (0.23-4.17)
	Neutral	78	132	37.1%	1.38 (0.35-5.49)
	Mostly trust	65	59	52.4%	2.57 (0.64-10.40)
	Completely trust	7	9	43.8%	1.81 (0.34-9.69)
Relying on AI chatbot use for hypothesis formation (*P* = 0.001)	Never (ref)	20	51	28.2%	-
	Rarely	52	82	38.8%	1.62 (0.87-3.02)
	Sometimes	59	97	37.8%	1.55 (0.84-2.85)
	Often	37	21	63.8%	4.49 (2.13-9.46)
	Always	7	8	46.7%	2.23 (0.71-6.97)
Willing to cite studies aided by an AI chatbot (*P* = 0.003)	Definitely no (ref)	6	17	26.1%	-
	Probably no	21	57	26.9%	1.04 (0.36-3.00)
	Maybe	70	111	38.7%	1.79 (0.67-4.75)
	Probably yes	63	58	52.1%	3.08 (1.14-8.34)
	Definitely yes	15	16	48.4%	2.66 (0.83-8.53)
Considering using an AI chatbot in future research (*P* < 0.001)	No (ref)	8	52	13.3%	-
	Maybe	39	118	24.8%	2.15 (0.94-4.92)
	Yes	128	89	59.0%	9.35 (4.23-20.64)
Thoughts about the evolution of AI chatbots in medical research in the next 5-10 years (*P* = 0.001)	Significant decrease (ref)	2	1	66.7%	-
	Decrease	1	1	50.0%	0.50 (0.01-19.56)
	No change	7	19	26.9%	0.18 (0.01-2.36)
	Moderate increase	46	112	29.1%	0.21 (0.02-2.32)
	Significant increase	119	126	48.6%	0.47 (0.04-5.28)
Declare the use of an AI chatbot for journals during submission (*P* = 0.468)	No (ref)	51	62	45.1%	-
	Maybe	57	88	39.3%	0.79 (0.48-1.30)
	Yes	67	109	38.1%	0.75 (0.46-1.21)

Awareness, perception, and attitude

Prior awareness of AI chatbots was strongly associated with use (users among the *aware*: 47.4% vs. 5.5% among the *unaware*; OR = 15.53, 95% CI 5.55-43.44; *P* < 0.001). Awareness of existing regulatory guidelines was also associated with higher use (56.4% vs. 34.4%; OR = 2.47, 95% CI 1.60-3.81; *P* < 0.001). The source of awareness (social media, friends, news, conferences, others) did not differ significantly (*P* = 0.073), as shown in Table 2.

Trust in AI chatbots showed a positive association with use (*P* = 0.005): respondents who *mostly trust* AI chatbots had higher odds of being users than those who *completely do not trust* them (OR = 5.33, 95% CI 1.10-25.75). A similar pattern held for trust in the results of AI-aided studies (*P* = 0.015). Frequent reliance on chatbots for hypothesis formation was concentrated among users (*P* = 0.001; *often *vs. *never *OR = 4.49, 95% CI 2.13-9.46). Willingness to cite AI-aided studies was higher among users (overall *P* = 0.003; *probably yes *vs. *definitely no *OR = 3.08, 95% CI 1.14-8.34). Willingness to future use was very strongly related to current use (*P* < 0.001; *yes *vs. *no *OR = 9.35, 95% CI 4.23-20.64). The intent to declare chatbot use during journal submission did not differ by user status (*P* = 0.468).

## Discussion

General potential uses of an AI chatbot

In late November 2022, LLMs drew substantial attention in the online world, including scientific publishing, because they can generate text that may appear similar to human-written content [14]. One major strength is their ability to process large amounts of text quickly, which may reduce researchers’ workload. ChatGPT and similar tools are trained on very large-scale text corpora and can support language-based tasks at scale, which is why they are often described among the largest language models in the world. These models also have the potential to automate tasks that were previously carried out manually [11]. For example, they may help users rapidly review academic papers and extract key elements (e.g., author names, publication dates, and main findings) [15-17]. More broadly, there is increasing interest in automating systems that can learn from data volumes that are too large to be handled efficiently by humans [18]. In 2023 and till 2025, the International Conference on Machine Learning adopted restrictions on AI-generated text in submissions, while allowing limited AI-assisted use under author responsibility, and clarified that LLMs are not eligible for authorship [19,20]. This reflects that parts of the research community support limited, responsible integration of AI tools into research workflows.

Certainly, AI chatbots may benefit authors when used responsibly. They cannot replace researchers’ subject-matter expertise, but they may assist with drafting or refining descriptions of findings and organizing manuscripts according to journal style requirements. AI chatbots may also support literature-related tasks, assist with aspects of analysis, and help generate or refine research questions. However, important risks include lack of context, inaccuracy, and bias in generated outputs [21,22]. For example, one study evaluated AI responses to 21 prompts related to dacryocystorhinostomy (DCR) surgery (e.g., history, anatomy, silicone intubation, and mitomycin C use) and reported factual inaccuracies in 60% of responses, with some answers on controversial topics being generic and not evidence-based. The authors also noted that the model may not consistently capture field-specific clinical details, which can lead to incorrect outputs [22].

Perception and attitude of researchers

In our sample, many participants reported that AI chatbots could have a high impact on medical research. Participants also reported moderate to high trust in AI-assisted outputs, while still expressing caution rather than complete reliance. Importantly, given the cross-sectional design, these findings should be interpreted as describing perceptions and reported behaviors, not as evidence that trust leads to use (or that use increases trust). Nevertheless, about 50% of participants indicated that they were considering using AI chatbots in the future, including both prior users and non-users. These findings are consistent with Eppler’s study, which reported that 47.7% of urologists used LLMs in academic practice and that most users expressed partial rather than complete trust [11]. This pattern may be reassuring because it aligns with the expectation that AI chatbot outputs require human oversight and that researchers may intentionally avoid complete trust to maintain accuracy. Additionally, 71.1% of participants believed that AI chatbot use requires ethical considerations, which may reflect awareness of potential risks and the need for responsible governance of AI-assisted research practices.

Acceptance of using an AI chatbot

ChatGPT, according to publishers and preprint servers contacted by Nature’s news team, does not meet authorship criteria because it cannot take responsibility for the content and authenticity of scientific studies [23]. Accordingly, AI use is generally expected to be disclosed during submission. Several major publishers have updated their policies to permit AI tool use when disclosed and when authors remain accountable. Large publishers (e.g., Springer, Elsevier, Taylor & Francis, Wolters Kluwer, and the Journal of the American Medical Association (JAMA) Network), which together account for more than 8,500 journals, require disclosure of AI authoring tools and emphasize author responsibility for all aspects of the manuscript [24]. The Science family of journals also updated editorial policies specifying that text generated from AI/LLM tools cannot be used in Science articles without explicit editor permission [25]. In our sample, 40.5% of participants indicated they would not declare AI chatbot use to journals during submission, including both users and non-users, suggesting potential gaps in awareness, adherence, or acceptance of disclosure expectations. Some journals also use AI-output detectors [26,27]. However, one study found that both scientists and AI-detection tools misclassified the origin of one-third of abstracts when comparing published abstracts versus ChatGPT-generated abstracts for the same articles [13]. This suggests that undetected AI-generated or AI-assisted text may be difficult to reliably identify in some contexts, reinforcing the importance of clear disclosure and policy awareness.

AI chatbot authorship

Authorship attribution is a key concern when LLMs contribute to research writing, alongside questions about ownership of generated content. If a user provides input data and the model generates output based on that input, one argument is that the user may hold rights over the generated content, though this remains a debated area [28]. The Committee on Publication Ethics (COPE) stated that “COPE joins organizations, such as the World Association of Medical Editors (WAME) and the JAMA Network, among others, to state that AI tools cannot be listed as an author of a paper” [29]. Springer has similarly stated that LLMs typically do not meet authorship standards because authorship requires accountability, which LLMs cannot provide; therefore, any LLM use should be clearly disclosed in the methods section or another appropriate section if a methods section does not exist [30].

Limitations and future implications

This study offers a brief look at the current status of AI chatbot use among researchers rather than cause-and-effect evidence, so we cannot tell whether using chatbots increases trust or whether trusting them leads to use. In addition, recruitment was conducted through non-probability convenience sampling using an online survey. The instrument was developed for this study and was not pilot tested or formally validated, so perception/attitude findings should be interpreted as exploratory. It would also be more helpful to include more details about the researchers, including their publication numbers, the type of studies, and more. The English-only, online, social-media recruitment likely introduced self-selection, language, and coverage biases, which limit generalizability beyond the four included countries. All data were self-reported and, therefore, vulnerable to recall and social-desirability biases despite anonymity. Future work should adopt more representative, multilingual sampling across regions. Longitudinal or panel designs can track how use, trust, disclosure, and policy awareness evolve as tools and guidelines mature.

## Conclusions

This study examined researchers’ characteristics and perceptions regarding AI chatbot-assisted studies. A substantial proportion of participants reported awareness of AI chatbots, and many, particularly younger respondents and those in training roles (e.g., students and interns), reported using them. AI chatbot use was associated with age and professional role: younger and less senior participants were more likely to report use and higher trust in these tools, whereas older or more senior professionals were less likely to report use and higher trust. In addition, participants who reported prior use also tended to report higher satisfaction. Notably, a considerable proportion of non-users reported willingness to use AI chatbots in the future. Overall, these findings suggest patterns of adoption and attitudes toward AI chatbots within our sample, and should be interpreted as exploratory associations rather than evidence of causation.

Although these findings are not generalizable beyond the study context, they highlight practical considerations for the responsible integration of AI chatbots in research settings. Efforts may benefit from improving researchers’ familiarity with AI chatbot capabilities, clarifying appropriate use-cases, and reinforcing transparent disclosure practices. Future studies should use more representative sampling and stronger designs to evaluate how use, trust, disclosure, and policy awareness evolve, and to inform clearer guidelines that support ethical and accountable use of AI-assisted tools in medical research.

## Figures and Tables

**Figure 2:**
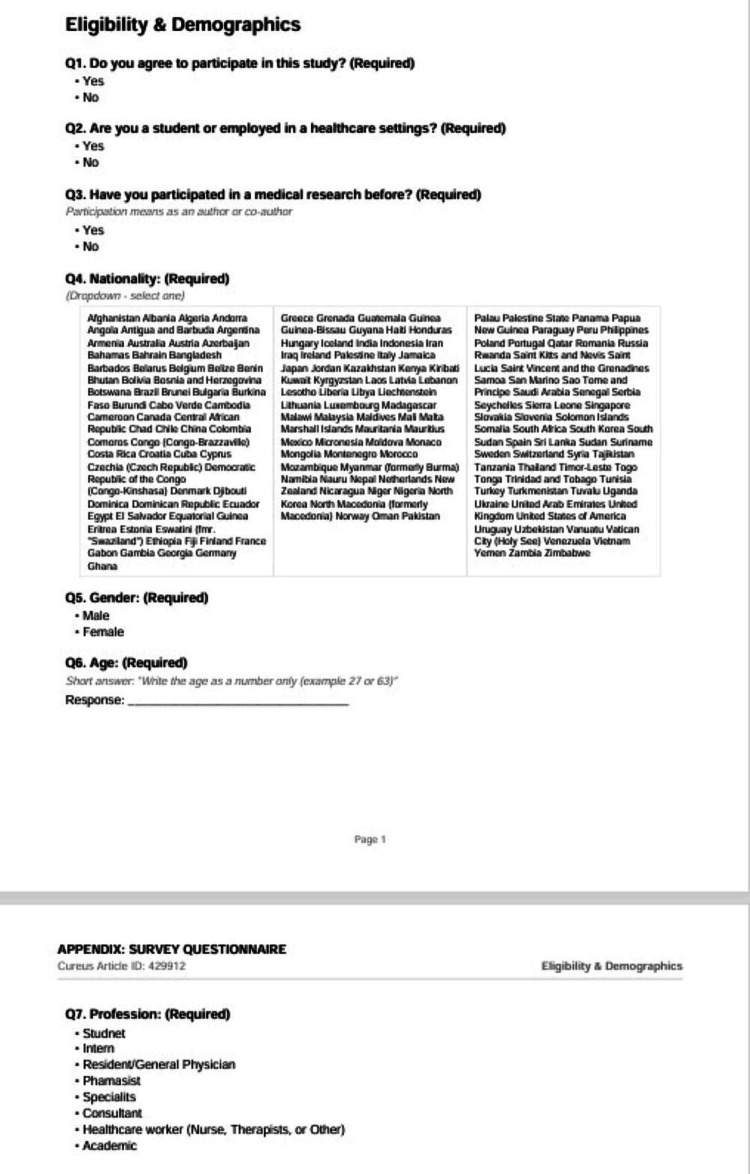
Eligibility and demographics.

**Figure 3:**
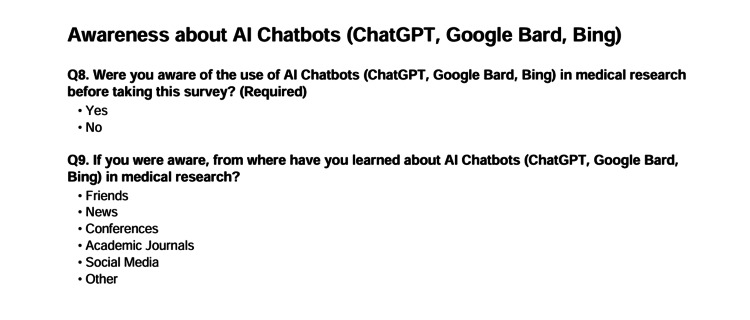
Awareness about AI chatbots.

**Figure 4:**
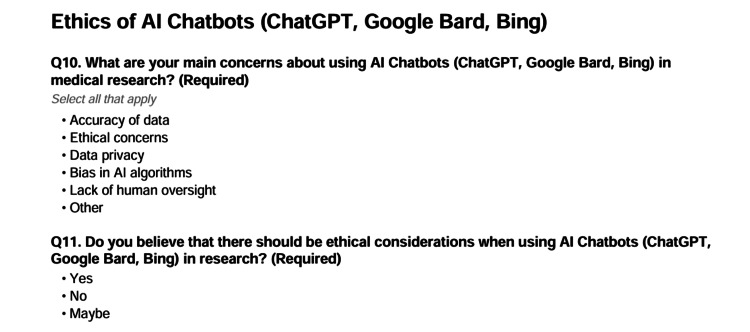
Ethics of AI chatbots.

**Figure 5:**
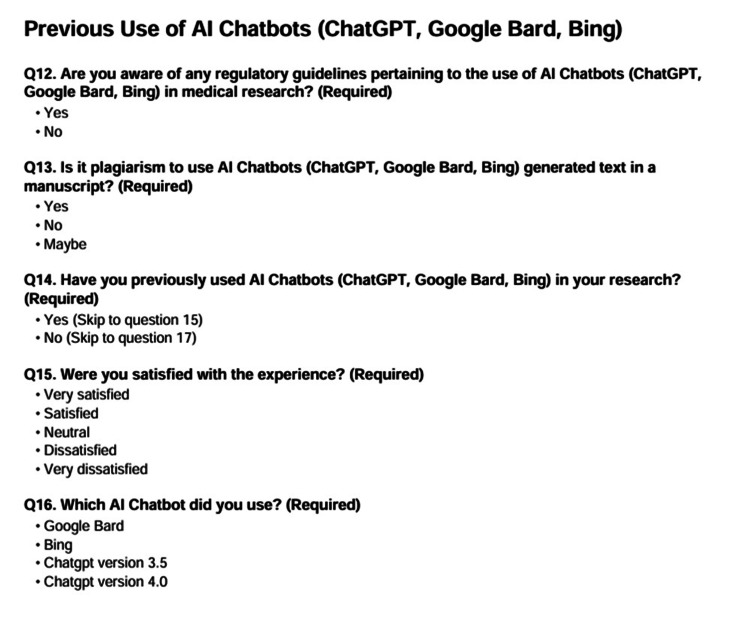
Previous use of AI chatbots.

**Figure 6:**
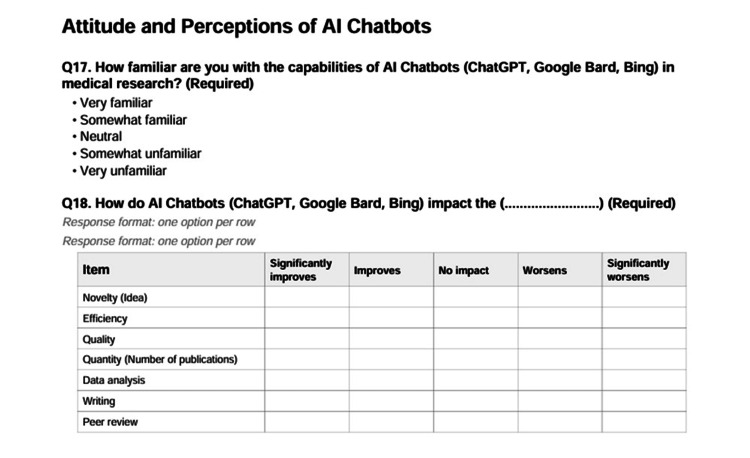
Attitudes and perceptions.

**Figure 7:**
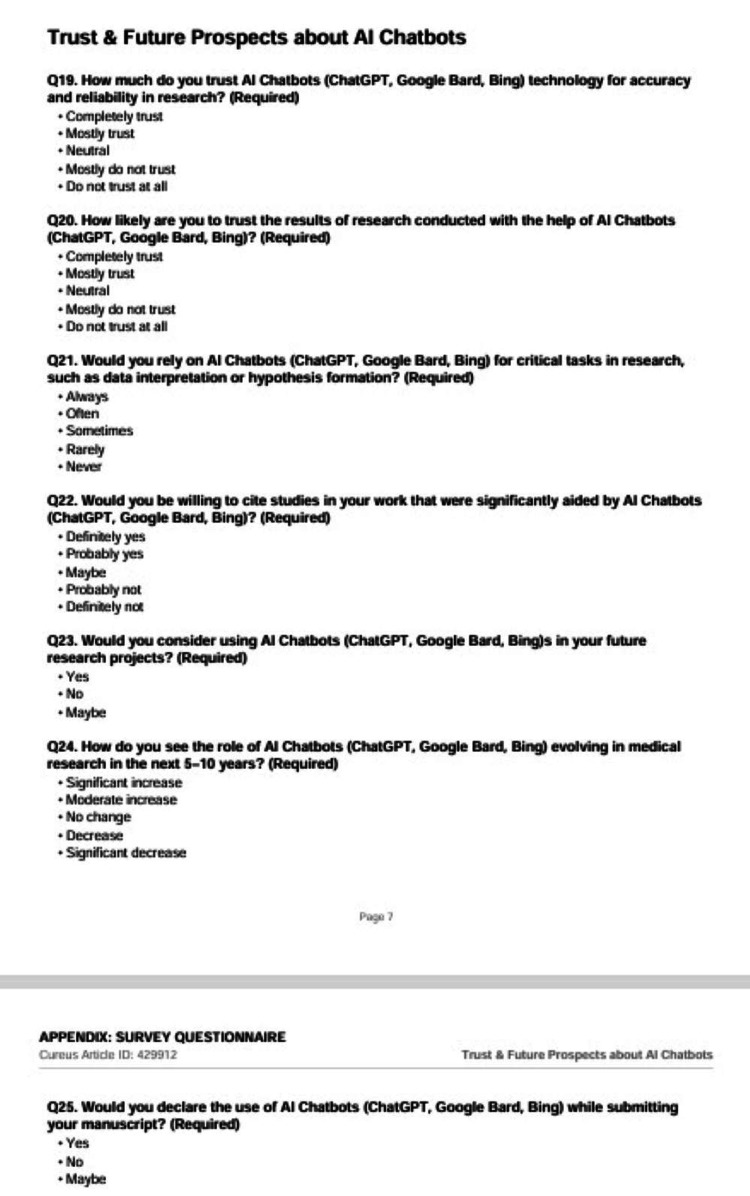
Trust and future prospects.

## Appendices

Appendix A

Appendix B

Appendix C

Appendix D

Appendix E

Appendix F
